# Comparative Metaproteomics and Diversity Analysis of Human Intestinal Microbiota Testifies for Its Temporal Stability and Expression of Core Functions

**DOI:** 10.1371/journal.pone.0029913

**Published:** 2012-01-18

**Authors:** Carolin A. Kolmeder, Mark de Been, Janne Nikkilä, Ilja Ritamo, Jaana Mättö, Leena Valmu, Jarkko Salojärvi, Airi Palva, Anne Salonen, Willem M. de Vos

**Affiliations:** 1 Department of Veterinary Biosciences, University of Helsinki, Helsinki, Finland; 2 Finnish Red Cross Blood Service, Helsinki, Finland; 3 Laboratory of Microbiology, Wageningen University, Wageningen, The Netherlands; Loyola University Medical Center, United States of America

## Abstract

The human intestinal tract is colonized by microbial communities that show a subject-specific composition and a high-level temporal stability in healthy adults. To determine whether this is reflected at the functional level, we compared the faecal metaproteomes of healthy subjects over time using a novel high-throughput approach based on denaturing polyacrylamide gel electrophoresis and liquid chromatography–tandem mass spectrometry. The developed robust metaproteomics workflow and identification pipeline was used to study the composition and temporal stability of the intestinal metaproteome using faecal samples collected from 3 healthy subjects over a period of six to twelve months. The same samples were also subjected to DNA extraction and analysed for their microbial composition and diversity using the Human Intestinal Tract Chip, a validated phylogenetic microarray. Using metagenome and single genome sequence data out of the thousands of mass spectra generated per sample, approximately 1,000 peptides per sample were identified. Our results indicate that the faecal metaproteome is subject-specific and stable during a one-year period. A stable common core of approximately 1,000 proteins could be recognized in each of the subjects, indicating a common functional core that is mainly involved in carbohydrate transport and degradation. Additionally, a variety of surface proteins could be identified, including potential microbes-host interacting components such as flagellins and pili. Altogether, we observed a highly comparable subject-specific clustering of the metaproteomic and phylogenetic profiles, indicating that the distinct microbial activity is reflected by the individual composition.

## Introduction

Following birth, our intestinal tract is colonized by an increasingly complex microbial community that is maintained from early to late adulthood [1]–[3]. The molecular characterization of the intestinal microbiota by phylogenetic approaches has received considerable attention in recent years and revealed a remarkable compositional stability and resilience in adult life, even after pervasive treatments such as the use of antibiotics [4]–[6]. The next level of sophistication is provided by metagenomic approaches that aim to systematically decode the genetic information of the intestinal microbiota. Having started with sequencing the intestinal metagenome of two individuals [7], the field of metagenomics has experienced rapid and expansive technological developments [8]–[10]. A recent hallmark study involving 124 individuals identified 3.3 million unique microbial genes and indicated that each of those individuals studied harbour over half a million microbial genes [10]. In addition, the number of sequenced single genomes of microbes residing in the human intestine is steadily increasing, with 30,000 new genes having been added recently [11]. These high-throughput analyses lay the groundwork for predicting the genetic potential of the intestinal microbiota. Moreover, the rapidly increasing catalogue of genes from intestinal origin provides a platform for high-throughput functional characterization [3].

Functional metagenomic approaches, such as transcriptomics and proteomics, aim to identify those genes that are expressed and may reveal the actual mechanisms by which microbes impact on the intestinal ecosystem. An initial comparative analysis of the faecal transcriptome from a pair of monozygotic twins revealed several thousands of highly expressed genes per subject, particularly those genes involved in carbohydrate metabolism [12]. Moreover, a recent comparative metatranscriptome profiling study of faecal microbiota showed highly individual gene expression that remains stable in time and includes dominantly expressed genes involved in carbohydrate metabolism [13]. Similarly, analysis of the intestinal transcriptome of Bifidobacteria from infants revealed significant stability of gene expression over a three-month period, but also specific response to diet [14]. Although transcriptome analysis is a powerful tool to determine gene expression, it relies on mRNA stability which is extremely low in prokaryotes [15]. Furthermore, mRNA transcripts do not necessarily represent the microbial function which is ultimately mediated by proteins. Given that proteins are much more stable than mRNA [16], proteome-based analyses can be expected to provide a better and more accurate view of the functionality of the intestinal microbiota. Initial metaproteomics analysis of faecal samples using two-dimensional gel electrophoresis highlighted the temporal development of the microbial proteins during the first days of infant life [17]. Moreover, a recent “snapshot” analysis of the intestinal metaproteome from an adult twin pair identified highly abundant proteins involved in translation and carbohydrate and energy metabolism [18]. This study used high-throughput mass spectrometric analysis in combination with the first available metagenomic database. Recently, we studied the metaproteome of two healthy individuals whose intestinal metagenome had been determined [19]. However, the differences between the intestinal metaproteomes of adult individuals, including any changes over time, have neither been addressed nor coupled to the microbiota composition.

Here we report the development of a high-throughput metaproteomics pipeline, which is applicable to large cohort sizes. This pipeline was benchmarked by comparing the intestinal metaproteome of unrelated subjects, who provided faecal material twice during a one-year period. After assessing the reproducibility of our approach by analysing the proteomic liquid chromatography-mass spectrometric (LC-MS) profiles, we compared the studied samples from a biological perspective and discovered the temporal stability and subject specificity of the intestinal metaproteome. Various proteins were identified by searching metagenome-based databases, revealing the presence of a common functional core that was mainly involved in sugar transport and degradation. This metaproteomics approach was complemented by phylogenetic array analysis that allowed us to couple functional properties to specific microbial groups.

## Results

### Main characteristics of the metaproteomic pipeline

**Figure 1:**
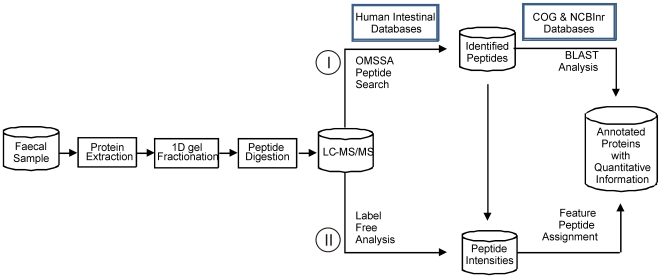
Metaproteomic analysis pipeline. Proteins were released from cells by mechanical disruption and then separated on a 1D gel prior to LC-MS/MS analysis. The MS/MS spectra were searched with OMSSA against databases (see Table 3) containing sequences to be expected in faecal material. All MS spectra were aligned over different runs and peptide identification was assigned to the detected features (route I). To functionally annotate the identified peptides and proteins, BLAST analyses were performed against the COG and NCBI non-redundant (nr) databases (route II).

**Figure 2:**
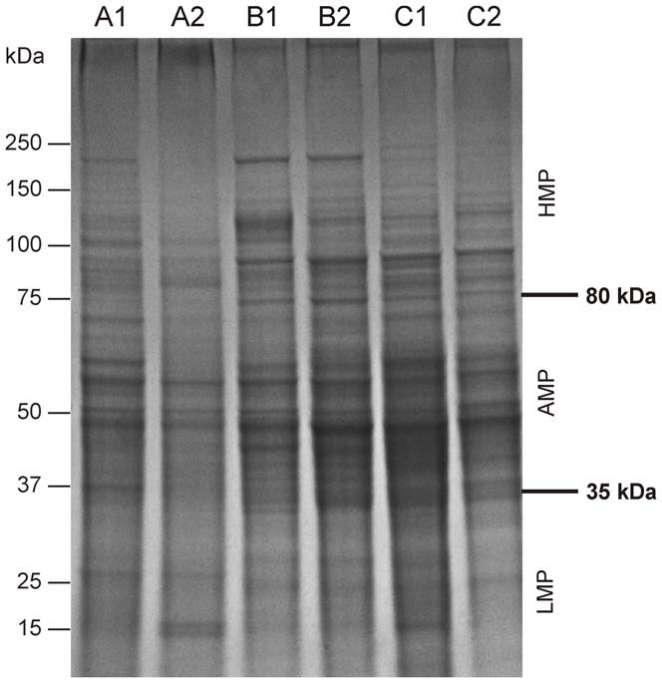
1D-Gel showing the protein pattern from faecal samples of three subjects at two time points. Estimated molecular weights are indicated on the left. Arrows on the right (upper arrow: 80 kDa, lower arrow: 35 kDa) indicate the locations where the gel was cut to obtain the Abundant MetaProteome (AMP, area between the cutting lines), the High Molecular MetaProteome (HMP, area above 80 kDa), and the Low Molecular Metaproteome (LMP, area below 35 kDa). Fractionation of the faecal metaproteome on gel visualized a within-subject similarity, i. e. temporal stability.

To functionally characterize the intestinal microbiota, we established a metaproteomic pipeline that can be used for rapid analysis of multiple samples (Figure 1). Unprocessed faecal material was used as a starting point not only to recover the microbial proteome, but also to allow detection of human and food proteins. Cells were physically disrupted since this was shown previously to be an effective method for accessing the recalcitrant microbes of the diverse intestinal microbial community [20]. Due to the heterogeneous composition of faecal material and the complexity of its metaproteome, specific sample purification and protein fractionation prior to liquid chromatography-tandem mass spectrometry (LC-MS/MS) analysis was required. We accomplished this simply and robustly by separating the extracted proteins using sodium dodecyl sulfate-polyacrylamide gel electrophoresis (SDS-PAGE) (Figure 2).

To develop a high-throughput metaproteomics workflow, that allows LC-MS measurements of dozens of different samples within a short time, we followed a minimal fractionation approach. Three fractions per metaproteome separated by using SDS-PAGE were generated and included (i) a high molecular weight metaproteome (HMP) fraction containing proteins larger than 80 kDa, (ii) a low molecular weight metaproteome (LMP) fraction with proteins smaller than 35 kDa, and (iii) a final fraction containing proteins in the range of 35–80 kDa. The latter was termed the Abundant Meta Proteome (AMP) fraction since it included the majority (81.4%) of identified proteins. The HMP and LMP fractions contributed to an additional 4.6% and 14.0% of the total amount of identifiable proteins, respectively (Table S1). Based on these results, we selected the AMP for the further use in validating our approach.

### Reproducibility and robustness of the pipeline

To validate our workflow, we performed a comparative analysis of the AMP fraction using feature-level data (i.e. m/z value per retention time and corresponding intensity) that was obtained with a software tool for label-free quantitative proteomics. Initially, we wanted to assure that the technical variation in the presented approach was sufficiently small to allow the detection of biological differences. Therefore, the effects of protein extraction, protein fractionation and gel cutting, and LC-MS analysis (Figure S1) and their reproducibility based on the feature intensities per run were used to calculate Pearson correlations (r) between samples.

To assess whether our gel-based approach was able to differentiate the technical, temporal, and subject effects, we calculated averaged sample similarities that were derived from two experiments. These included faecal samples from up to three healthy and unrelated subjects (subjects A, B and C) were obtained from two time points and repeatedly analysed (Table 1, see Material and Methods section). The results showed that the sample preparation effect was significantly smaller (r = 0.66) than the subject effect (r = 0.39). Moreover, samples from different subjects were more dissimilar than the two time points from a single individual (r = 0.51). This high technical reproducibility allowed drawing biological conclusions and the application of the approach to further studies as described below.

**Table 1:** Technical and biological variation in the faecal metaproteomic analysis of subjects A, B and C.

Variation	A, B (SD; n)	A, B, C (SD; n)	Average (SD; n)	Confidence
Experimental	0.72 (0.06; 50)	0.58 (0.05; 35)	0.66 (0.06; 85)	p<0.05
Temporal	ND	0.51 (0.04; 34)	0.51 (0.04; 34)	p<0.05
Subject	0.25 (0.04; 54)	0.41 (0.03; 156)	0.39 (0.07; 210)	p<0.05

Sample similarities are expressed as Pearson correlations, which were calculated for peptide feature intensities between different sample preparation replicates (experimental), different time point (temporal), and subjects (A, B, C) (ND: not determined; SD: standard deviation; n: number of comparisons). Column “A, B” presents data from an experiment in which the metaproteome of subjects A and B time point one were analysed and column “A, B, C” shows data of a repetition of those samples and an addition of a second time point and subject C. For detailed experimental setup see “Material and Methods” Figure S1.

### Individuality and temporal stability of the intestinal metaproteome

**Figure 3:**
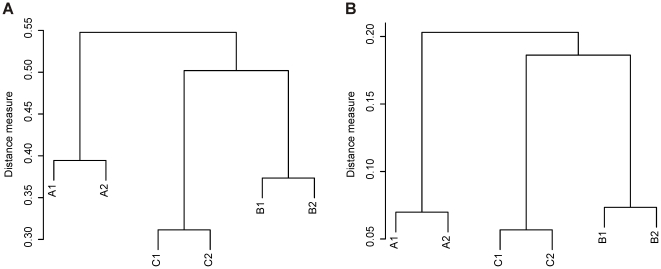
Hierarchical clustering of mass spectrometric (A) and phylogenetic fingerprints (B). Distance as 1-Pearson correlation values are indicated on the vertical axes. Subject-wise clustering implies that the proteomic (averaged over the measurements per sample in set of 22 AMP measurements; see Figure S1) and the phylogenetic fingerprints remain characteristic for each subject over a period of one year.

**Figure 4:**
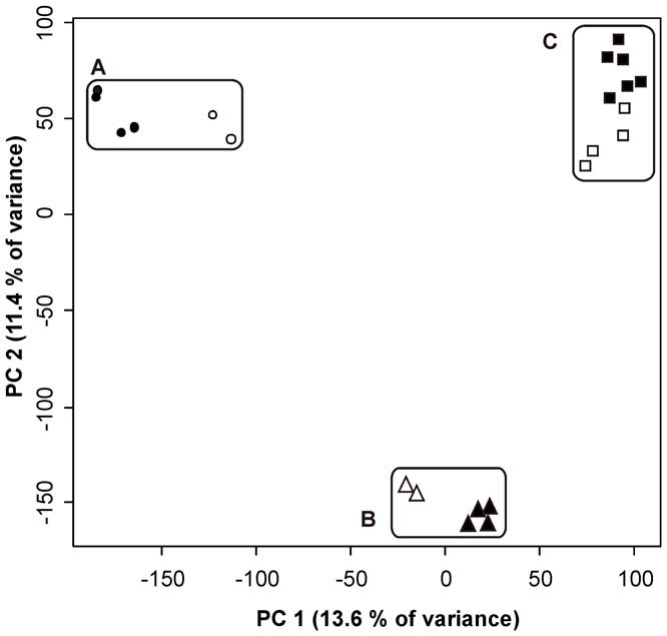
PCA plot visualizing separation of metaproteomic data according to technical-replicates, time points and subjects. Feature intensities of 22 LC-MS runs were plotted according to the first two principal components. Technical replicates of the first time point are presented in grey and measurements from the second time point are in black.

The similarity of the faecal metaproteomes from three subjects was determined over time to establish its temporal stability. Hierarchical clustering of the averaged log intensities of 26,000 features detected in 22 AMPs (Figure S1) revealed a subject-specific metaproteome (Figure 3A). It was also evident that each subject had its characteristic metaproteome that was retained over a period of 6–12 months. This unsupervised clustering supported visual observations made after protein staining, which had revealed clear subject-specific patterns and, to a lesser extent, variation over time (Figure 2). To further extend these observations we subjected the data to a Principal Component Analysis (PCA) (Figure 4). Based on 26,000 data points, the resulting PCA plot revealed a clear separation between the subjects (PC1) and time points (PC2), both for log intensities of the features and the abundances of assigned proteins. The top two principal components (out of 22) together described 25% of the total variance.

### Identification of microbial proteins and metabolic pathways in the intestinal metaproteome

A total of over 100,000 spectra were generated by the collection of MS/MS from 37 AMP, 11 HMP, and 11 LMP measurements. From these, we could identify 1,790 proteins with two or more peptide identifications (Table S1). To retrieve further functional information, we annotated these proteins based on the Cluster of Orthologous Groups (COG) classification that is used to functionally classify bacterial and archaeal proteins [21]. A total of 164 different COGs could be assigned to over 70% of the identified proteins. To obtain the common microbial core proteome, we selected the proteins that were found in all the three studied subjects at least in one time point (see Material and Methods). A total of 1,216 proteins fulfilled this criterion and based on the spectral counts the majority of these could be grouped into 25 COGs (Table 2). The most salient features of the microbial core proteome are described below.

**Table 2:** The 25 most abundant COGs of the protein core detected in human intestinal samples.

COG group	Definition	COG functional category	MS/MS Spectra [%]*
COG0334	Glutamate dehydrogenase/leucine dehydrogenase	E	14.3
COG1866	Phosphoenolpyruvate carboxykinase (ATP)	C	6.6
COG1653	ABC-type sugar transport system, periplasmic component	G	5.2
COG2115	Xylose isomerase	G	4.7
COG0057	Glyceraldehyde-3-phosphate dehydrogenase/erythrose-4-phosphate dehydrogenase	G	4.1
COG0176	Transaldolase	G	3.5
COG0574	Phosphoenolpyruvate synthase/pyruvate phosphate dikinase	G	3.5
COG0183	Acetyl-CoA acetyltransferase	I	3.3
COG0148	Enolase	G	2.0
COG3957	Phosphoketolase	G	2.7
COG0126	3-phosphoglycerate kinase	G	2.5
COG1882	Pyruvate-formate lyase	C	2.2
COG0166	Glucose-6-phosphate isomerase	G	2.0
COG2160	L-arabinose isomerase	G	2.0
COG0174	Glutamine synthetase	E	1.9
COG0021	Transketolase	G	1.6
COG0059	Ketol-acid reductoisomerase	EH	1.2
COG0480	Translation elongation factors (GTPases)	J	1.2
COG1454	Alcohol dehydrogenase, class IV	C	1.2
COG0282	Acetate kinase	C	1.2
COG1250	3-hydroxyacyl-CoA dehydrogenase	I	1.1
COG2407	L-fucose isomerase and related proteins	G	1.1
COG1904	Glucuronate isomerase	G	1.0
COG0822	NifU homolog involved in Fe-S cluster formation	C	1.0
COG0459	Chaperonin GroEL (HSP60 family)	O	0.9

*contribution in percent of specific COG group to the total amount of identified spectra in the core metaproteome.

COG functional categories: C Energy production and conversion; E Amino acid transport and metabolism; G Carbohydrate transport and metabolism; H Coenzyme transport and metabolism; I Lipid transport and metabolism; J Translation, ribosomal structure and biogenesis; O Posttranslational modification, protein turnover, chaperones.

**Figure 5:**
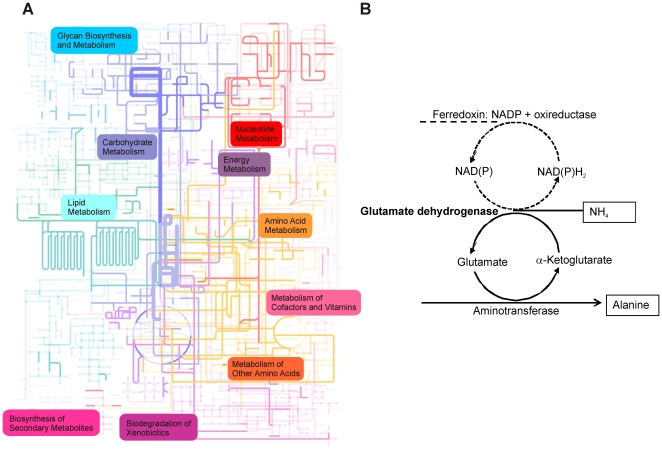
Represented metabolic pathways in the human intestinal metaproteome. A Visualization of all identified metabolic pathways. The pathways “Glycolysis and Gluconeogenesis” (blue) and “Pyruvate Metabolism” (purple) for which most enzymes were identified are highlighted. B Glutamate dehydrogenase reaction. According to Kengen et al. glutamate dehydrogenases may couple amino acid transfer to electron transfer [22]. Glutamate is transformed to alpha-ketogluratae by the consumption of NAD(P) and the assistance of aminotransferases. Ferredoxin-oxidoreductase reduces NAD(P)H_2_ to NAD(P).

The most abundant core protein was found to be glutamate dehydrogenase (GDH) to which the largest number of peptides could be mapped. Moreover, detailed analysis of these peptides revealed GDH to show a high level of redundancy in the intestinal tract since we could identify it as a major protein in a large variety of bacterial families, including the *Lachnospiraceae*, *Bacteroidaceae*, *Ruminococcaceae* and *Bifidobacteriaceae* (Table S1, Table S2). It is known that GDH links the nitrogen and the carbon-cycle via the incorporation of ammonia into 2-ketoglutarate. However, GDH may also have another metabolic role and act as an electron sink (Figure 5). This pathway, which operates in several strict anaerobes to assure a low level of free electrons, results in the net conversion of pyruvate and ammonia into alanine while consuming NAD(P)H that can be generated via a ferredoxin NAPDH oxidoreductase [22]. This potential new role of intestinal GDH as an electron sink requires the activity of aminotransferases, many of which were identified in the metaproteome, including the ornithine/acetylornithine aminotransferase, phosphoserine aminotransferase, and aspartate/tyrosine/aromatic aminotransferase (Figure 5). Remarkably, the other well-known electron sink is the formation of lactic acid from pyruvate via lactate dehydrogenase. However, this pathway appears to be less efficiently operating in the colon, since with only 14 spectra the well-conserved bacterial lactate dehydrogenase was found in much less amounts than GDH (Table S1). In addition, a remarkably high number (4%) of the identified spectra could be assigned to phosphoenolpyruvate carboxykinase, a gluconeogenic enzyme conserved both in Gram-positive and Gram-negative bacteria.

Another abundant core protein was annotated as pyruvate-formate lyase (Table 2). This oxygen-sensitive enzyme is widely spread in anaerobes and converts pyruvate to acetyl-CoA and formate. In the presence of formate-hydrogen lyase, the formed formate is converted into hydrogen and CO_2_, whereas the acetyl-CoA is converted into acetate by acetate kinase with the formation of ATP. Both formate-hydrogen lyase and acetate kinase were identified with a high number of peptides (56 and 13 peptide hits, respectively) in the core metaproteome, indicating that this important energy-generating metabolic pathway operates in the intestinal ecosystem.

Additionally, among the top 25 COGs of the common core was GroEL, a chaperonin belonging to the HSP 60 family that is involved in protein folding. Another core protein with chaperone function is the NifU homolog, a protein involved in Fe-S cluster formation [23]. This is to be expected since many of the anaerobic enzymes contain Fe-S clusters. For example, pyruvate∶ferredoxin oxidoreductase is involved in the oxidative decarboxylation of pyruvate into acetyl- CoA and is an alternative for pyruvate-formate lyase while generating reduced ferredoxin that is an important electron carrier in anaerobes. Nearly 1% of all the spectra of the common core could be assigned to pyruvate∶ferredoxin oxidoreductase, indicating the importance of this pathway and the need for effective Fe-S cluster formation.

Compatible with the important sugar degradation potential of the gut microbiota, various proteins involved in carbohydrate transport and metabolism were identified as part of the core metaproteome (Table 2). This group accounted for over 10% of the total proteome and included ABC sugar transporters and glycolytic enzymes, such as 3-phosphoglycerate kinase, which assists in the transformation of 3-phospho-D-glycerate to 3-phospho-D-glyceroyl phosphate by consumption of ATP. The abundance of bacterial proteins devoted to the utilization of carbohydrates testifies for their importance as metabolic substrates in the intestinal tract.

To address the higher functional level of all the 1,790 identified proteins, we mapped the identified COGs onto Kyoto Encyclopedia of Genes and Genomes (KEGG) pathways. One of the most predominant pathways was glycolysis, showing high redundancies among the phyla; nine glycolytic/gluconeogenic enzymes along with eight accessory enzymes could be identified. This finding highlights the importance of glucose as metabolic substrate of the human intestinal microbiota. The identified KEGG pathways were then mapped on a global metabolic pathway map (Figure 5). This mapping revealed that the functional categories “Carbohydrate Metabolism”, “Nucleotide Metabolism”, “Energy Metabolism”, and “Amino Acid Metabolism” are most predominant, reflecting the high metabolic activity of the intestinal microbiota. Since this finding held true for all the faecal samples, we could expand on single-time point observations made previously [18] and presume that the listed functional categories are constantly highly abundant in healthy subjects. The identification of the KEGG group “Metabolism of Cofactors and Vitamins” that includes the notable presence of proteins involved in vitamin B12 and folic acid synthesis in all subjects, testifies for one of the essential functions of the intestinal microbiota [10].

### Comparison of functions over time and between the subjects based on AMP

Beside the common functional core, we were interested in the inter-individual and temporal differences at a functional level. Therefore, we visualized the abundance of the 960 proteins, identified with two or more peptides, representing 22 AMPs to visualize the differences between the subjects and the time points (Figures S1, S2, S3). In the cluster with the highest abundances in all subjects there were, for example, GDH, xylose isomerase, and glutamine synthetase. The subject-wise clustering observed on peptide feature level could be repeated at a protein and COG function level (data not shown), indicating stable expression of the identified proteins. Hence, not only protein intensities, but also abundance of the particular functional classes appeared to be subject-specific. To utilize all the identification information, identified spectra from all (Figure S4A) and only AMP (Figure S4B) measurements were used to illustrate the characteristic functional distribution per individual and time point. Several of the major COGs showed marked differences between the subjects and time points that are further discussed below in relation to the microbial compositional changes.

### Identification of surface proteins

Of specific interest were those proteins that allow the intestinal microbes to interact with one another and with the human host. Several different flagellins were identified as part of the core metaproteome in all samples. Especially for subject A many identifications for flagellins were observed (4% of all the identified spectra) which is approximately 10-fold more than for subjects B and C. Moreover, when protein sequences that had no hit in the COG database were subjected to a BLAST search against the National Center for Biotechnology Information (NCBI) non redundant database, we obtained several hits with high confidence (E-value ranging between 1.0E^−04^ to 0.0) for various classes of surface proteins, including those for pili and surface-layer proteins (Table S2). Notably, seven pilus-like proteins were found in subjects B and C, six of which were pilus-like proteins from Streptococci, which may derive from the ileum where such species are abundant [24].

### Non-bacterial proteins

The vast majority of the identified proteins, approximately 90%, were of microbial origin. A single food protein was identified and derived from rice (a chloroplastic pyruvate phosphate dikinase), thus confirming the efficiency of the intestinal tract for digesting food. Moreover, in total, 45 human proteins, excluding possible contaminants [25], were identified as representing host cell activity along the digestive tract (Table S1).

### Phylogenetic characterization of the studied microbiota

To assess whether the subject-specific metaproteome differences were related to the compositional differences of the microbiota, and to generally describe the microbes that had potentially produced the identified proteins, we studied the microbial composition of the samples analysed here. Faecal DNA was extracted, by lysing cells in the same mechanical way as done for the metaproteome analysis, and subjected to phylogenetic analysis with the Human Intestinal Tract Chip (HITChip) microarray, which can detect over 1,000 unique bacterial phylotypes [26]. The most predominant bacteria in the studied subjects belonged to the Firmicutes, covering 70% (C2) to 95% (B1) of the total microbiota. The most abundant Firmicutes were the members of Clostridium cluster XIVa and Clostridium cluster IV, each comprising an average proportion of 58% and 26% of the total microbiota, respectively. The relative share of the phylum Bacteroidetes ranged from 3% (B1) to 26% (C2), and that of Actinobacteria from 1% (B1 and C1) to 3% (C2).

Hierarchical clustering of the microarray signals from the faecal DNA revealed that the microbial fingerprints from the same individual from the two different time points were more similar to each other than to those from the two other subjects (Figure 3B). Hence, both the phylogenetic and the metaproteomic data resulted in a clustering with an identical topology, highlighting the subject-specificity of both data types (Figure 3). The overall bacterial composition remained highly similar within a subject during the follow-up period (mean r = 0.94). We did not observe differences in the degree of microbiota similarity between the samples that were collected half a year (r = 0.94; subject C) or a year apart (r = 0.94; subject A and 0.93; subject B). Zooming in on individual taxa revealed substantial variation in their proportional share in all samples (Table S3), which reflects the individuality and temporal dynamics of the microbiota. For example, the most dominant genus-level taxon, *Faecalibacterium prausnitzii* and relatives (*et rel.*), had a 3-fold decrease in subject A and almost 1.5-fold decrease in subject B from the first to the second time point. Subject C and particularly sample C2, varied the most from the other subjects due to its substantially higher abundance of Bacteroidetes and Actinobacteria. On the other hand, the dominant intestinal archaeon *Methanobrevibacter smithii* was not found in subjects B and C, but was abundant in samples from both time points of subject A (mean of 6.1×10^9^ 16S RNA copies g^−1^ faeces).

### Comparison of proteomics and compositional data

The relation between metaproteome levels and the microbial composition was studied with a random forest classifier, which predicted the protein levels using the phylotype level data from the HITChip analysis as covariates ([27]; see Materials and Methods). In total, the model identified 50,091 significant protein-phylotype associations (p<0.05). To increase the reliability of the predictions and facilitate the interpretation of the results, the phylotype-level associations were further enriched to genus level using a Fisher exact test. As a result, 3,999 significant associations (p<0.05) were found (Figure S5). For example, the flagellin proteins were associated with species of the groups *Roseburia intestinalis* and *Eubacterium rectale*. This was confirmed by BLAST searches of the respective sequences, identifying representatives of both species (Table S3). The same holds true for the detected association between alpha-glucosidase and the group *Ruminococcus obeum*. Additionally, a visual inspection of the changes on proteins and microbial species revealed interpretable links between the abundance of certain bacterial groups and the expression of proteins. We detected changes in the amount of the ABC sugar transporters (high in samples A1 and C2 and low in samples A2 and C1) that were in the same direction as those of *F. prausnitzii et rel.* in the HITChip data (high in samples A1 and C2, low in samples A2 and C1). This analysis was partly supported by the random forest classifier, which found a significant connection between *F. prausnitzii* and COG1653, a ubiquitous ABC-type sugar transporter. In total, nine proteins had significant association at the genus level to *F. prausnitzii et rel.*, a group of abundant butyrate producers belonging to the Firmicutes. The total number of significant associations at phylotype-level was 104. However, with COG1879, another ABC-type sugar transporter, only one significant phylotype-level association was found. A BLAST search of the ABC transporter sequences from both COGs indicated highest similarity to proteins derived from *F. prausnitzii*. Furthermore, clear differences in the amount of identified spectra for phosphoenolpyruvate synthase were observed, which might have been caused by *Ruminococcus obeum*-like species as the presence of this bacterial group and this gluconeogenic enzyme followed clearly the same trend in samples from subjects B and C. The high amount of phosphoenolpyruvate synthase in subject A might derive from *F. prausnitzii*, which is present in high amounts in this subject. If so, the distribution of phosphoenolpyruvate synthase testifies for the functional redundancy of this energy generating system in the intestinal microbiota. These observations were supported by the random forest analysis which found 38 significant associations between phosphoenolpyruvate synthase and *R. obeum et rel.*, and 30 significant associations between the enzyme and *F. prausnitzii et rel.*

**Figure 6:**
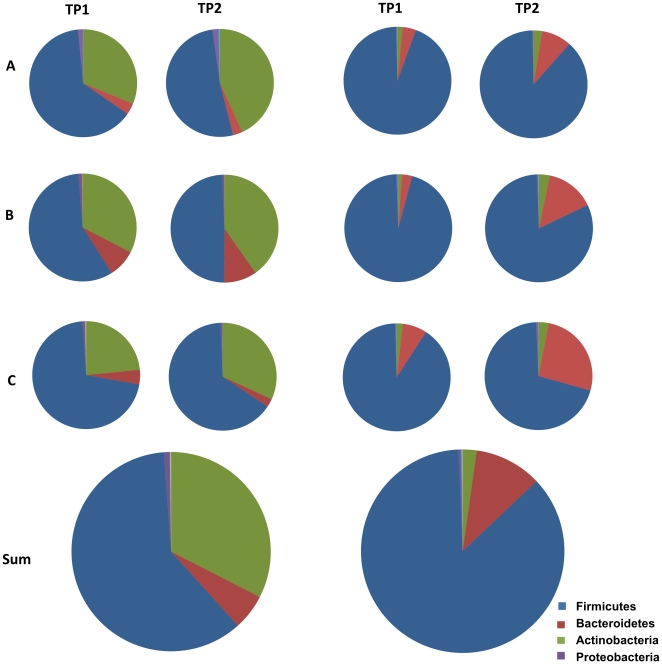
Phylogenetic classification based on metaproteome and phylogenetic analysis. The major phyla per subject (A, B, and C) and time point (TP1 and TP2) based on metaproteome (left) and phylogenetic (right) analysis summed and averaged over all analysed samples are presented.

Although deeply rooted and therefore subject to reliable phylogenetic assignment, we did not detect proteins specifically derived from *Methanobrevibacter*. This genus represented only 0.3% of the total microbiota in subject A and was absent from the others, reflecting the fact that the metaproteome approach reveals only proteins that derive from the abundant microbiota. Similarly, low levels of *Akkermansia* spp. were found in all subjects and hence only a few proteins could be assigned to this unique intestinal member of the Verrucomicrobia [28], although it was highly abundant in an earlier studied metaproteome [19]. For other less deeply rooted taxa, it is difficult to assign an origin to specific proteins. However, by using the UniProt Knowledgebase (UniProtKB) we were able to classify about half of the identified spectra at least into bacterial phyla (Figure 6). The metaproteomic assignment was compared with that observed by analysing the bacterial composition of the same sample by using the phylogenetic microarray (Figure 6). The majority (60%) of the assigned spectra was found to derive from the phylum Firmicutes. This is in line with the phylogenetic microarray analysis that showed this phylum to be the most abundant one (86%). *F. prausnitzii* was the most abundant representative of this phylum in both the metaproteomic and phylogenetic analyses. In contrast, the high proportion (33%) of peptides from Actinobacteria differs from the phylogenetic analysis (only 2%). Consequently, the relative share of Bacteroidetes was lower in the peptide (6%) than in 16S rRNA data (11%). About 0.2% of the peptides with at least a phylum assignment could be attributed to the Proteobacteria, which is approximately 10-fold lower than their abundance (2%) observed with the phylogenetic microarray, while only a few spectra derived from the phyla Fusobacteria, Chlamydiae/Verrucomicrobia group, Spirochaetes, Deinococcus-Thermus, and Synergistetes. The distribution of the peptides with a phylogenetic assignment deeper than Bacteria was compared between the samples (Figure 6). The same trends in the changes of the phyla between the two time points could be observed at the metaproteomic and 16S RNA level. In subjects A and B the contribution of Firmicutes decreased, whereas Bacteroidetes and Actinobacteria increased with time. For subject C the trends between metaproteomic and 16S RNA data were less coherent. Only a decrease of Firmicutes could be consistently observed with both approaches.

**Figure 7:**
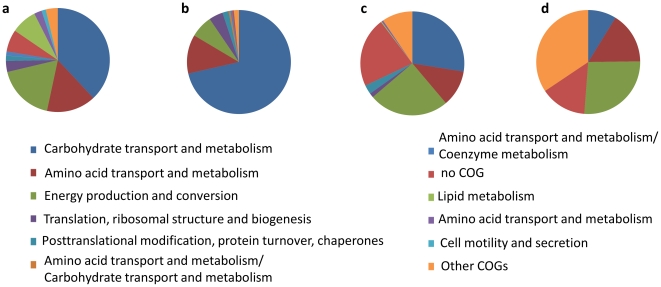
COG distribution per phylum. The COG functional category distribution per phylogenetic group, based on the peptide measurements, is visualized. a) Firmicutes, b) Actinobacteria, c) Bacteroidetes and d) Proteobacteria.

To illustrate the differences in functionalities between the four main phyla Firmicutes, Actinobacteria, Bacteroidetes, and Proteobacteria the spectral hits for each COG functional category per phylum were counted (Figure 7). Our metaproteomic data confirmed earlier observations that the Firmicutes showed an active carbohydrate metabolism and butyrate production [29], [30]. Close to 75% of the identified actinobacterial peptides were predicted to be involved in sugar metabolism confirming the dominant role in transforming carbohydrates by this phylum [31]. The metabolic distribution of Bacteroidetes showed more mixed functions with less dominant abundance of enzymes involved in carbohydrate metabolism, energy production and amino acid metabolism [32]. Notably, almost 25% of the peptides assigned to Bacteroidetes could not be classified in any COG and hence represent functions that need to be further unravelled.

To verify the presence of a trophic chain we could identify in the same sample lactate dehydrogenase from Bifidobacteriales and acetyl-CoA acetyltransferase from *Eubacterium hallii*, indicating the production of butyrate via lactate [33].

## Discussion

Metaproteomics compared to other functional genomics approaches such as transcriptomics has the advantage that actual and stable gene products can be studied. Since the physico-chemical and phylogenetic composition of faecal material is more complex than other environmental samples (e. g. waste water) metaproteomic investigations of the human intestinal microbiota are still in a pioneering phase [34]–[36]. In this paper we demonstrate the robustness of a new metaproteomic pipeline with high-throughput features. The developed approach does not involve any purification of the bacterial cells and for this reason is fast and comprehensive already at an early stage of sample preparation. To increase the efficiency and throughput of our approach we focused our attention on a region that included the majority of bacterial proteins, the 35–80 kDa AMP. We showed that analysis of the AMP was sufficient to reveal differences between the individuals and the stability of the individual metaproteome over time. However, some proteins with high relevance for intestinal function, such as signalling peptides, might remain outside the targeted region and thus require tailored methodologies to be characterized.

We benchmarked our approach by describing the intestinal metaproteome of three healthy subjects using faecal samples taken twice within a period of one year. Typically, studies of only short duration (few weeks or months) are carried out when investigating the intestinal microbiota. We chose a longer period for faecal sampling and revealed that the changes in the proteins observed over a one-year interval were smaller than observed between the metaproteomes of different subjects. We also provided a deep and global analysis of the microbiota of the same intestinal samples by using the HITChip microarray, which permitted us to compare the temporal dynamics of species and protein composition. As a novel finding, we observed that the microbiota function, as reflected by the metaproteome, shows a significant temporal stability. Remarkably, this metaproteome stability is comparable to that of the microbial composition, which has been addressed previously [26], [37]. This indicates that the function and composition of the intestinal microbiota are strongly coupled.

The 1,790 identified proteins described here, only capture a portion of the predicted coding capacity of the intestinal microbiome [10]. The actual expression level of the predicted genes and their redundancy at the proteome level is not known. Similar to the next generation sequencing technologies, the depth of analysis affects the “coverage” of the complexity of the intestinal microbiota in metaproteomic studies. Additionally, with the present databases, which are already more than 10-fold larger than described previously, we only could assign about 15% of the detected spectra [18]. However, higher identification ratios can be expected in the future since our insight about the intestinal microbial complexity and advances in high quality metagenome sequencing are increasing rapidly [11]. The increase in protein identification rate based on a matched metagenome, a one of-the-kind result, was shown recently [19]. Taken together, our work demonstrates that a relatively shallow approach with a high-throughput pipeline is capable of identifying the major functionalities in the intestinal tract. Moreover, it enabled us to reveal subject-wise differences in the microbiota composition that mimic those observed using deep analysis techniques (Figure 3). Consequently, data from shallow molecular analyses of the intestinal microbiota appear suitable for screening purposes, whereas deeper analyses are required for cataloguing purposes [19]. This is a similar approach as has been suggested recently for studying phylogenetic differences between individuals with new generation sequencing approaches [38]. With that said, we suggest that our protocol might encourage also other fields applying metaproteomic approaches to focus on a relevant subfraction of the sample in order to facilitate screening of large sample sets with less analytical resources.

Based on the proteins present in all subjects, we could identify a common proteome core that included many glycolytic enzymes, proteins involved in pyruvate metabolism, chaperones and other stress proteins, and proteins associated with vitamin biosynthesis. These protein activities are compatible with functions of the intestinal microbiota as deduced from metagenomic studies [7]–[10]. Similarly, the identification of high amounts of proteins involved in carbon and nitrogen metabolism such as ABC sugar transporters, GDH, pyruvate-formate lyase and phosphoenolpyruvate carboxykinase, is consistent with the observations in previous metagenomics and metatranscriptomics analyses. In a recent study, a single pair of twins was analysed at the metagenome and metatranscriptome level and revealed high expression of the genes for ABC transporters, GDH and phosphoenolpyruvate carboxykinase in both siblings [12]. In our study, we observed a high abundance and strikingly large isoformic variation among the GDHs in the analysed samples. In addition, one of the first comprehensive metagenomic studies [8] showed that pyruvate-formate lyase (COG1882) was abundantly present at the gene level and predicted to be the most abundant protein of PfamA families in the human intestine [39]. Phosphoenolpyruvate carboxykinase was also described as being overrepresented in the human gut metagenome compared to other genomes [8]. Moreover, we could also identify in high amounts all the proteins discussed in an earlier metaproteomics study of a Swedish twin pair [18], namely rubrerythrin, formyltetrahydrofolate synthetase, fucose metabolism proteins, butyryl-CoA dehydrogenase, and NifU-like homologs.

Surface proteins of the intestinal microbiota are of special interest since they enable the microbes to interact directly with host cells. We identified several different flagellin proteins in all samples, especially in subject A. However, the phylogenetic microarray analysis did not indicate large differences between the samples in the abundance of the dominant flagellated bacteria, *Roseburia* and *Butyrivibrio spp.*, between the samples. Hence, the inter-individual difference in the amount of identified flagellins probably reflects their actively regulated expression. The highly conserved bacterial flagellins are known to signal to Toll-like receptor 5 and to be involved in innate and adaptive immune responses [40]. Additionally, the intestinal flagellins may provide the microbes with the ability to be motile, allowing them to better reach their food sources as has been suggested for *Roseburia intestinalis* and *R. inulinivorans*, both of which are luminal bacteria that can degrade complex carbohydrates [41]. Remarkably, flagellin genes were depleted in an earlier comprehensive metagenome library [8], but this might be as a consequence of the way the comparative analysis was performed [39]. Increased antibody levels towards flagellins have been identified in Crohn's disease and post-infective IBS patients, suggesting that flagellins are medically relevant protein markers of the intestinal microbiota [42]. By using bioinformatic approaches we were also able to detect other surface proteins, which included pili and surface-layer proteins, both known to interact with or signal to host cells [43], [44].

Besides the microbial proteins, we identified 45 non-redundant human proteins. This is somewhat lower than what was found in an earlier study, which however included multiple isoforms [18]. In general, the identified human proteins are involved in digestion of carbohydrates and proteins, immune reactions for defence and tolerance, maintaining mucosal barrier function, and providing an energy source for microbes. One example is amylase, which is one of the very first secreted enzymes to digest carbohydrates by being already present in the mouth. The detection of amylase in the faecal samples suggests its synthesis in the lower digestive tract, which is much in line with the suggestion that amylase is also produced in cells other than parotid and pancreatic cells [45].

To link the production of proteins to the abundance of specific microbes, we performed random forest analyses, plain trend comparisons between the metaproteome and phylogenetic data, as well as phylogenetic assignment of the peptides. The random forest analysis suggests a large number of significant associations between the bacteria and proteins. Most of the associations are currently difficult to interpret, in part due to the functional redundancy and conservation of bacterial proteins that tends to hamper their reliable assignment to a particular species. Moreover, it is known that there is a strong individual response to diet and other environmental conditions [46].

At a phylum level, the inter- and intra-individual differences in the phylogenetic composition of microbial communities derived from the 16S rRNA and the metaproteome analyses yielded the same trend. However, there was a prominent difference in the relative share of Actinobacteria of the metaproteome and phylogenetic array analyses. Many peptides appeared to derive from Actinobacteria, specifically Bifidobacteria, while these were not abundant in the phylogenetic analysis. One possible explanation could be that Bifidobacteria are highly active while not numerically dominant. High activity of specifically Bifidobacteria was already suggested based on RNA/DNA ratios in healthy humans [47], after prebiotic diet [48] and following analysis of the live, injured and dead fractions of the faecal microbiota by flow cytometry [49]. Similarly, in a previous metaproteomics study [18], a high number of hits were found within the genus Bifidobacterium when searching spectra against a database that included a set of 34 bacterial genomes. Besides the apparent high activity of Bifidobacteria, other possible explanations include the stability of bifidobacterial proteins as well as the overrepresentation of these proteins in databases or their highly conserved nature. In addition, many of the here identified proteins could be assigned to *F. prausnitzii*-like bacteria. This corresponds well with the compositional analysis as Faecalibacteria are among the most abundant genera colonizing the healthy intestinal tract. Moreover, this group was also among the taxa having a high RNA/DNA signal [47]. In a previous work, it has been reported that Bacteroidetes sequences are overestimated in metagenomics data [12]. However, this might be due to incomplete databases, since we could not confirm this by using UniProtKB, which is a much larger reference database. The congruency of the here described functionalities per phyla and the earlier described functionalities are a further validation of our approach.

In conclusion, our multi-sample metaproteomics approach described here, allowed us to reliably identify faecal proteins that are stable, abundant, and conserved. Hence, our findings provide supportive evidence for the presence of a substantial functional core of proteins involved in sugar transport and degradation as well as a large set of other proteins, many of which reflect the adaptation to the intestinal environment.

## Materials and Methods

### Faecal samples

Faecal samples from three healthy, omnivorous female subjects (A, B and C) in the age-range of 23–35 were collected in-house twice within a year (for A and B the sampling points were a year apart, for C half a year) and directly stored at −80°C. This study did not qualify as a clinical trial so that no ethical permission was required. The volunteers had provided written informed consent.

### Protein Extraction

Faecal samples were thawed on ice and homogenized prior to protein extraction. Proteins were mechanically extracted from the homogenized and unprocessed faecal material by bead beating in the following way: to 125 mg faecal sample 375 µl of 1× PBS, 500 mg 0.1 mm zirconium–silica beads (Biospec Products), 3 glass beads and a proteinase inhibitor (Roche) were added. The samples were covered with gaseous nitrogen. Five cycles of bead beating were run through with a FastPrep FP120 (Savant) at 6.5 ms−1 for 45 sec with 1 min cooling steps on ice in-between [17]. The resulting supernatants were subjected to low speed centrifugation to remove beads and high speed centrifugation to remove cell debris and then subsequently stored at −80°C.

### 1 D gel electrophoresis and in-gel protein digestion

To reduce the complexity of the protein extract a 1D gel fractionation according to molecular weight was carried out. Equal volumes of protein solutions were mixed with 4× Laemmli buffer and Biorad reducing agent and run on NuPAGE 4–12% Bis-Tris gels (Invitrogen) at 200 V. Gels were stained with PageBlue (Fermentas). The 37 kDa and the 75 kDa band of a prestained marker (Biorad) was used to define the height for cutting the lane into three pieces. For easier sample handling only one vertical half of the 35–80 kDa region of the separated metaproteome was used for in-gel protein digestion. Gel pieces were washed and proteins reduced, alkylated and tryptically digested overnight as described elsewhere [50].

### Experimental setup

In this study six different faecal samples called A1, A2, B1, B2, C1 and C2 were analysed. The letter indicates the subject from which the sample derived and the number the order of the sampling. The experimental setup is visualized in Figure S1 and includes the processing steps (protein extraction, protein fractionation and in-gel protein digestion). First, to test for the robustness of the pipeline, proteins from faecal samples A1 and B1 were extracted in triplicate. In addition, several replications of the protein fractionation, in-gel protein digestion and LC-MS analysis were conducted. The AMP was measured by LC-MS, in most cases in duplicate. From this first experiment 15 AMP measurements resulted. Second, individual and temporal differences were determined in the samples A1, A2, B1, B2, C1, and C2. Including various replications, 22 AMP measurements were conducted. A total of 59 LC-MS measurements were performed that included 37 AMPs, 11 LMP and 11 HMP measurements.

### LC-MS/MS measurements

Protein digests were analysed with LC-MS/MS. Peptides were loaded to a reversed phase precolumn (NanoEase Atlantis dC18, 180 µm×23.5 mm, Waters) with 0.1% formic acid and separated in a reversed phase analytical column (PepMap 100, 75 µm×150 mm, Dionex Corporation) with linear gradient (4–50%) of 95% acetonitrile in 0.08% formic acid in 85 minutes. The Ultimate 3000 liquid chromatography instrument (Dionex Corporation) was operated in nano scale with a flow rate of 0.3 µl/min. Both pre-column and analytical column were placed in a column oven at 30°C. Eluted peptides were introduced to the LTQ Orbitrap XL mass spectrometer (Thermo Fisher Scientific Inc.) via ESI Chip interface (AdvionBioSciences Inc.) in positive-ion mode.

The mass spectrometer was calibrated with Thermo Fisher Scientific standard LTQ calibration solution consisting of caffeine, MRFA tetrapeptide and Ultramark 1621. The instrument was tuned with glu-fibrinopeptide B (Sigma-Aldrich). Full scan for eluting peptides was acquired in the mass range of 300–2,000 m/z on Orbitrap-detector with 60,000 resolution at 400 m/z, AGC target set to 200,000 and maximum inject time set to 800 ms. Based on full scan, six MS/MS data-dependent scans were acquired on LTQ with AGC target set to 10,000 and maximum inject time set to 100 ms. Isolation width of 2 m/z was used for precursor selection. Normalized collision energy of 35%, activation time of 100 ms and activation Q set to 0.25 were used in peptide fragmentation. Precursors, whose charge state could not be determined or for which the charge state was +1, were discarded from MS/MS analysis. Precursors were dynamically excluded for 10 s with repeat count of 1. Both full scan and MS/MS scans consisted of one microscan and they were acquired as profile data.

When studying individual and temporal differences (Figure S1B), exclusion lists were used for the technical replicates. Precursors fragmented in the first measurement were listed to create an exclusion list and used for the technical replicates to prevent fragmentation of already fragmented peptides thus increasing the number of identifications.

### Differential proteomics analysis

LC-MS/MS Thermo .raw files were uploaded to Progenesis LC-MS 2.5 (Nonlinear Dynamics). Noise at a retention time <10 min and >60 min was excluded prior to automatic alignment of LC-MS runs and detection of features. Ions with <2 charges and <1 isotope were not considered for further analyses. The two data sets described above, consisting of 15 and 22 runs respectively (Figure S1), were analysed separately due to the unavoidable too large shift in retention time.

Protein identification results obtained using OMSSA [51] (see below) were imported to Progenesis LC-MS with a customised OMSSA identification importer from Nonlinear Dynamics.

### Database construction

Five in-house databases were created from five publicly available sequence databases: a selection of 298 human intestinal microbes (sources: ftp://ftp.ncbi.nih.gov/genomes/Bacteria/and/Bacteria_DRAFT/; date of download: 2010/4/24, Table S4), “European Metagenome MetaHIT” (source: http://www.bork.embl.de/~arumugam/Qin_et_al_2009/), “Japanese Metagenome” (source: ftp://ftp.ncbi.nlm.nih.gov/genbank/wgs/), a human protein database containing non-redundant proteins from Integr8 and Genbank (source: http://www.ebi.ac.uk/integr8/FtpSearch.do?orgProteomeID=25 and ftp://ftp.ncbi.nih.gov/genomes/H_sapiens/protein/; date of download: 2010/08/11) and a food database consisting of *Solanum lycopersicum* (tomato), *Oryza sativa* (rice), *Zea mays* (corn), *Malus domesticus* (apple), *Triticum aestivum* (wheat), *Vicia faber* (bean) and *Glycine max* (soybean) (source: http://www.uniprot.org/; date of download 2010/08/29). MetaHIT, the largest metagenomic dataset, consists of DNA sequences from intestinal microbiomes of 124 European individuals. The database European metagenome MetaHIT was created with the predicted proteins (minimum length: 33 AA) as provided by Qin et al. [10]. The resulting proteins were filtered for the presence of at least one fully tryptic peptide of a size of at least 5 AA. Gene prediction of the Kurokawa et al. reads [8] was done in the same way by using the prokaryotic gene-finding program MetaGene [52]. See Table 3 for the final number of proteins in each of the five databases. Of all the sequences within one database reverse sequences (rev) were generated and concatenated with the forward sequences (fwd).

**Table 3:** Databases used for protein identification.

Database name	Size (number of proteins)
European Metagenome MetaHIT	3,267,604
298 human intestinal microbes	947,087
Japanese Metagenome	600,752
Food database	247,371
Human database	69,879

### Protein identification

For peptide identification the Open Mass Spectra Search Algorithm OMSSA [51] was used to search MS/MS spectra against the above described databases. The used parameters were carbamidomethylation of cysteine (fixed modification), oxidation of methionine (variable modification) and one missed cleavage was allowed. The precursor mass tolerance was 0.03 Da and the product ion tolerance 0.4 Da. Otherwise default settings were applied. All five concatenated in-house databases were searched separately. If one spectrum matched different peptide sequences in different databases, the spectrum was re-searched against the suggested sequences and the best hit reported and taken for further analysis. The false discovery rate (FDR) from the concatenated database searches was calculated as follows: 2*hits in rev/hits in fwd and rev sequences [53]. The hits were filtered with a FDR of 5% and the results from the searches against the five different databases combined. Identified peptides were searched against all the sequences for which at least one peptide was identified and all possibilities were collected. Unique proteins were identified by grouping peptides. A protein was defined to be a unique count when at least one identified peptide was different to another sequence. Proteins identified with the same peptides, or proteins for which no other peptides than a subset of these peptides were identified, were clustered and counted as one hit. Proteins which were only identified with peptides that formed a sub-group of another protein were not considered to be unique.

In Table S1 the protein name is listed for which sequence most peptides were identified. If there were several sequences with exactly the same number of identified peptides, the sequence name from the 298 human intestinal microbial genomes database was chosen. The group number indicates that each of the proteins within that group shares at least one of the identified peptide with another protein in that group.

### Functional annotation

For the annotation of identified protein sequences, we used the Cluster of Orthologous Groups database (COG, [21]). Protein sequences were scanned against the COG database using BLAST [54]. The COG associated with the best BLAST hit (≤1E-10 cut-off) was assigned to the query protein. The identified COGs were mapped on the KEGG metabolic pathways and visualized with the online application of iPath [55]. Sequences which did not hit any specific COG were subjected to BLAST search against the NCBI non-redundant database.

### Defining of COG core

Microbial proteins identified in 37 AMPs, 11 HR and 11 LR and those, which had at least one peptide identification in each individual, were defined to build the core. The spectra those proteins were identified with were taken to sum on COG level per all measurements and were used to rank the COGs according to spectra number.

### Phylogenetic mapping of peptides

All identified peptides, after FDR correction, were mapped against UniProtKB. For each 100 per cent match (taking into account the tryptic peptide rule by OMSSA [51]) the taxonomical information was retrieved (Table 3) and the deepest shared level of taxonomic specification was determined. The species annotation was applied to all identified spectra and the counts were summed up to phylum level. All peptides with their phylogenetic assignment are shown in Table S5.

### Phylogenetic analysis

Bacterial DNA was extracted from the faecal samples with Repeated Bead Beating (RBB) method as described previously [20]. Compositional analysis of the microbiota was performed using the Human Intestinal Tract Chip (HITChip), a phylogenetic microarray produced by Agilent technologies (Agilent Technologies). Microarray analyses and computational pre-processing of the arrays was performed as previously described [26] and the references therein. The presence of archaeal DNA was analysed by using primers specific for *Methanobrevibacter* genus as previously described [20].

### Statistical data analysis

All statistical analyses were performed with in-house R scripts. Log-transformed data was used for all analyses except the relative quantification of the HITChip genus-level taxa.

Pearson correlation coefficients of features were calculated within the two sets of AMPs separately (15 and 22 respectively). Per test group (sample preparation, variation in time, variation between subjects) correlation coefficients were summed and averaged per LC-MS experiment (Figure S1) and combined (Table 1). An ANOVA test was applied to all correlation coefficients. Means of feature intensities/protein abundances were used to calculate Pearson correlations and cluster them hierarchically using complete clustering criterion. Principal component analysis [56] was applied to the set of 22 AMPs. Run-duplicates of each faecal sample were used to perform ANOVA analysis of regulated proteins/COGs.

HITChip data was analysed using Pearson correlation and hierarchical clustering, linear models and ANOVA tests [57]. Hierarchical clustering was used to group the samples according to correlation distance and complete clustering criterion.

### Associations between metaproteome levels and the microbial composition

Analysis was carried out in R version 2.12.1 using the randomForest package. Each of the 960 detected protein levels was predicted by random forest [27], using the 1,033 phylotypes detected by HITChip as covariates. For each protein, the variable importances detected by random forest were Z-scored, and those variables having a Z-score>1.96 (corresponding to being significantly different from zero with p-value<0.05) were chosen as the significant covariates. The phylotype-level connections were enriched to genus-like level by computing their enrichment with Fisher exact test (with significance level<0.05). Finally, the protein level results were summarized to associations to COG functional categories by computing the number of significant associations between the proteins in the category and the enriched genus-like taxa.

## Supporting Information

Figure S1 **Experimental setup.** A. Setup to test robustness of metaproteomic analysis pipeline, resulting in 15 AMP LC-MS/MS measurements used for analysis. B. Setup to test for individual and temporal differences, resulting in 22 AMP LC-MS/MS measurements.
(DOCX)

Figure S2 **Protein intensities per sample.** Protein intensities in abundant metaproteome AMP over three subjects and two time points. Triangles indicate the region in the heatmap where the respective protein is found. Red scale: intensities ≥10^5^; white: intensity ≤10^5^.
(PDF)

Figure S3 **Core metabolic pathways.** The metabolic pathways that are shared by all the subjects are shown on an iPath map.
(PDF)

Figure S4 **Functional comparison.** A compositional view of the most abundant COGs (based on MS/MS identifications over all measurements, A; and over all AMP measurements, B) is shown. From left to right the bars visualize the COG distribution of all subjects and time points (all), the core, the subjects (A, B, C) and for each time point per subject (A1, A2, B1, B2, C1, C2).
(TIF)

Figure S5 **Heatmap of significant associations between metaproteome and HITChip data.** Each row represents a genus-level taxon and each column represents a COG group. The colour key indicates the log10 of the number of significant associations between proteins and the enriched genus level groups.
(PDF)

Table S1 **List of identified proteins.**
(XLSX)

Table S2 **List of sequences with annotation “pili” and “surface-layer protein.”**
(XLSX)

Table S3 **Distribution of genus-level taxa per sample analysed with phylogenetic array.**
(XLSX)

Table S4 **List of human intestinal gut microbes (Genbank download 2010/4/24).**
(XLSX)

Table S5 **Peptides with phylogenetic assignment.** Interest in the raw data should be addressed to the corresponding author.
(XLSX)
