# Supplementary material for: Comparative Metaproteomics and Diversity Analysis of Human Intestinal Microbiota Testifies for Its Temporal Stability and Expression of Core Functions
Source: PLoS One. 2012 Jan 18;7(1):e29913. doi: 10.1371/journal.pone.0029913 (PMC3261163; doi:10.1371/journal.pone.0029913)
Supplement: Figure S1 — Experimental setup. A. Setup to test robustness of metaproteomic analysis pipeline, resulting in 15 AMP LC-MS/MS measurements used for analysis. B. Setup to test for individual and temporal differences, resulting in 22 AMP LC-MS/MS measurements. (DOCX) [file pone.0029913.s001.docx]

**Figure S1. Experimental setup. A.** Setup to test the robustness of metaproteomic analysis pipeline, resulting in 15 AMP LC-MS/MS measurements used for analysis. **B.** Setup to test for individual and temporal differences, resulting in 22 AMP LC-MS/MS measurements.


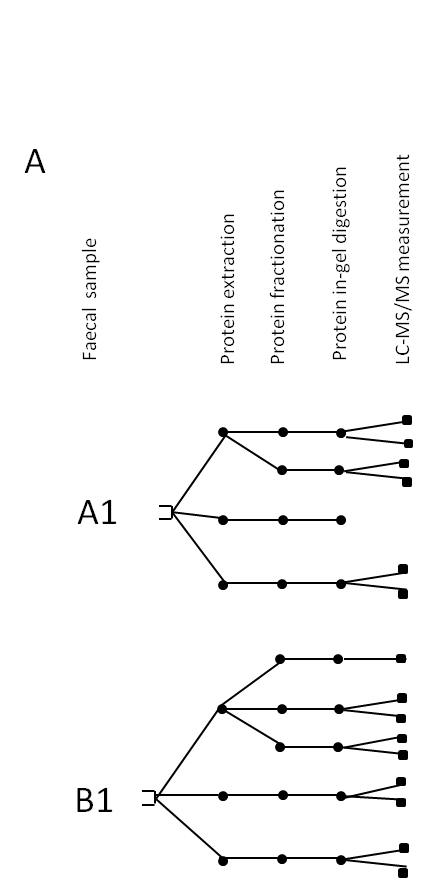


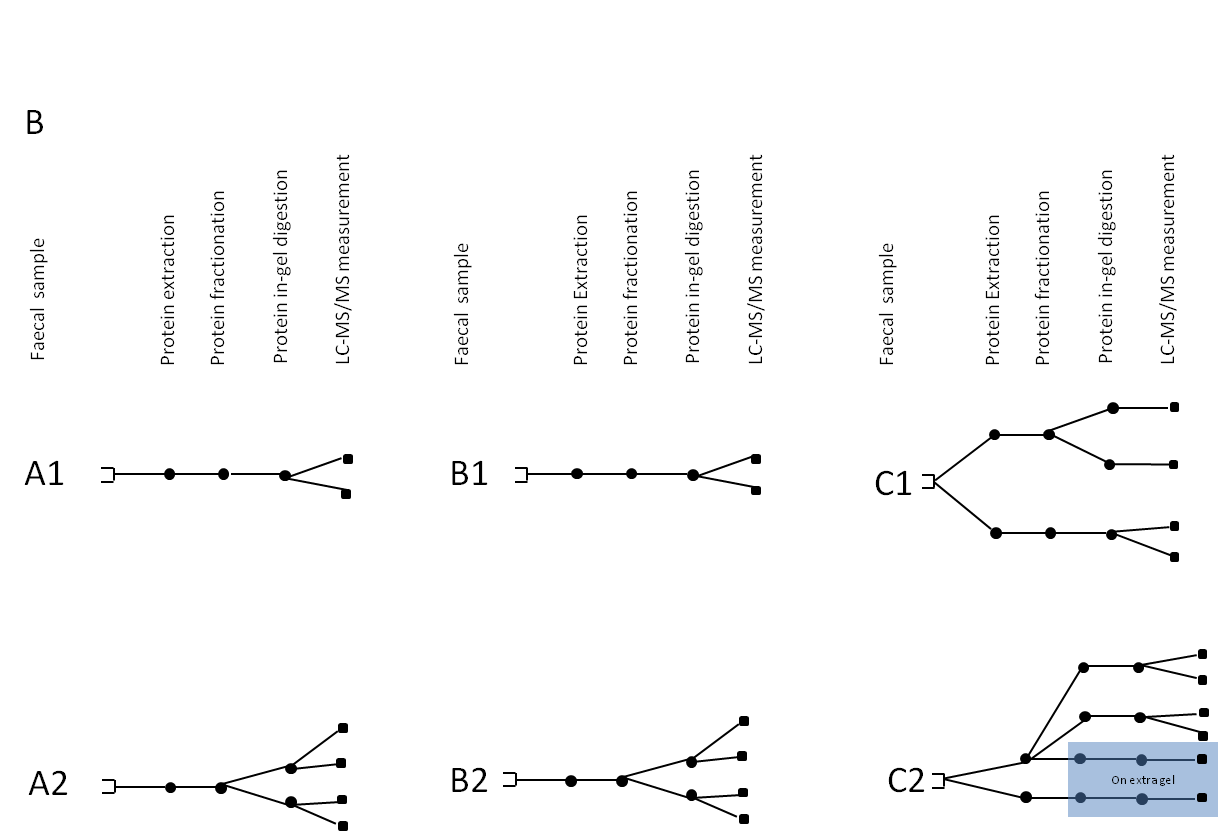


A1, A2, B1, B2, C1, C2: Faecal material from subjects A, B, and C at sampling time point 1 or 2; Open square: starting material; dot represents a procession step (protein extraction, protein fractionation and in-gel digestion); branch indicates splitting of the sample after the respective sample preparation step; filled square represents LC-MS/MS measurements.
