# Supplementary figures and images for: Comparative Metaproteomics and Diversity Analysis of Human Intestinal Microbiota Testifies for Its Temporal Stability and Expression of Core Functions
Source: PLoS One. 2012 Jan 18;7(1):e29913. doi: 10.1371/journal.pone.0029913 (PMC3261163; doi:10.1371/journal.pone.0029913)

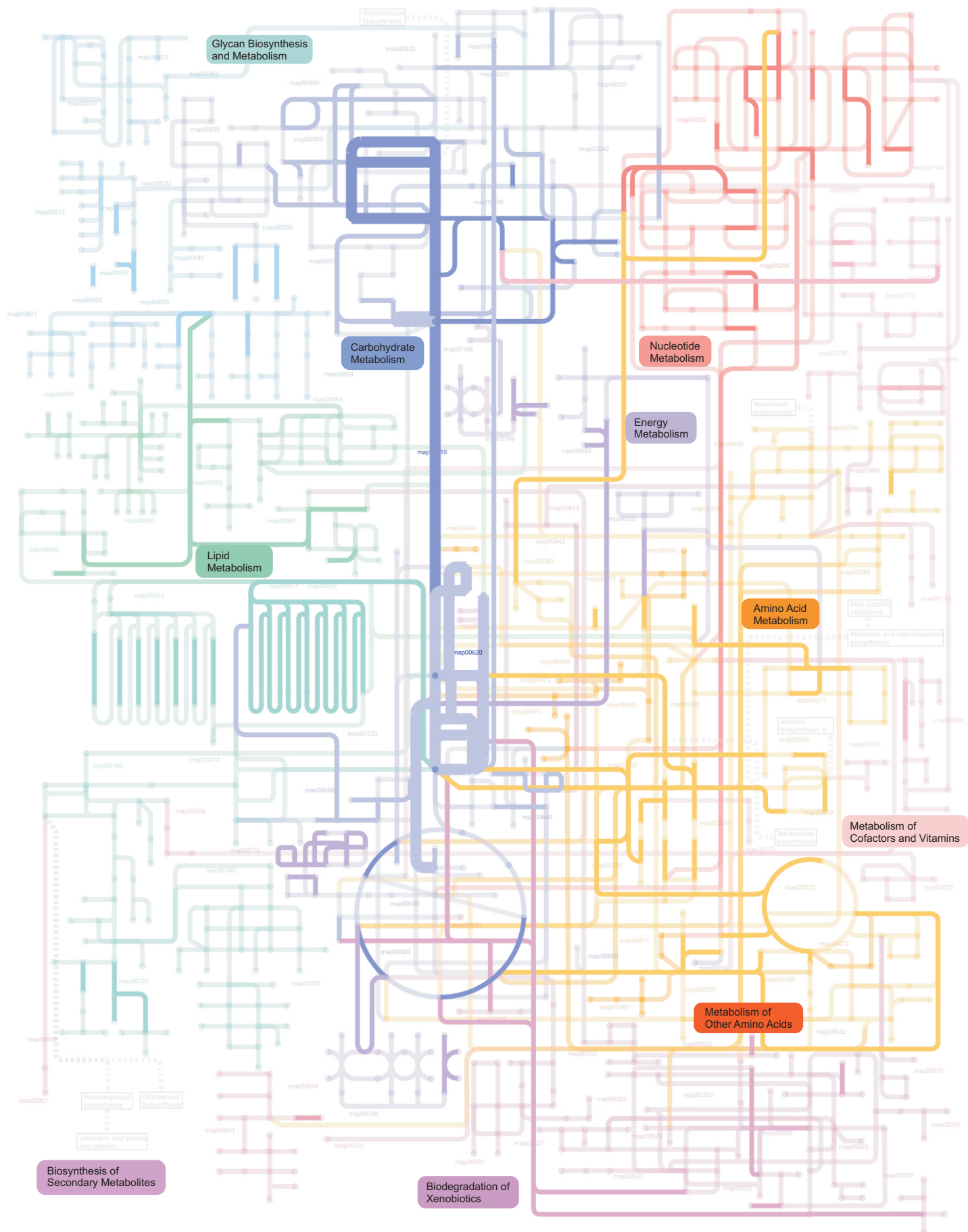

Supplement: Figure S3 — Core metabolic pathways. The metabolic pathways that are shared by all the subjects are shown on an iPath map. (PDF) [file pone.0029913.s003.pdf]

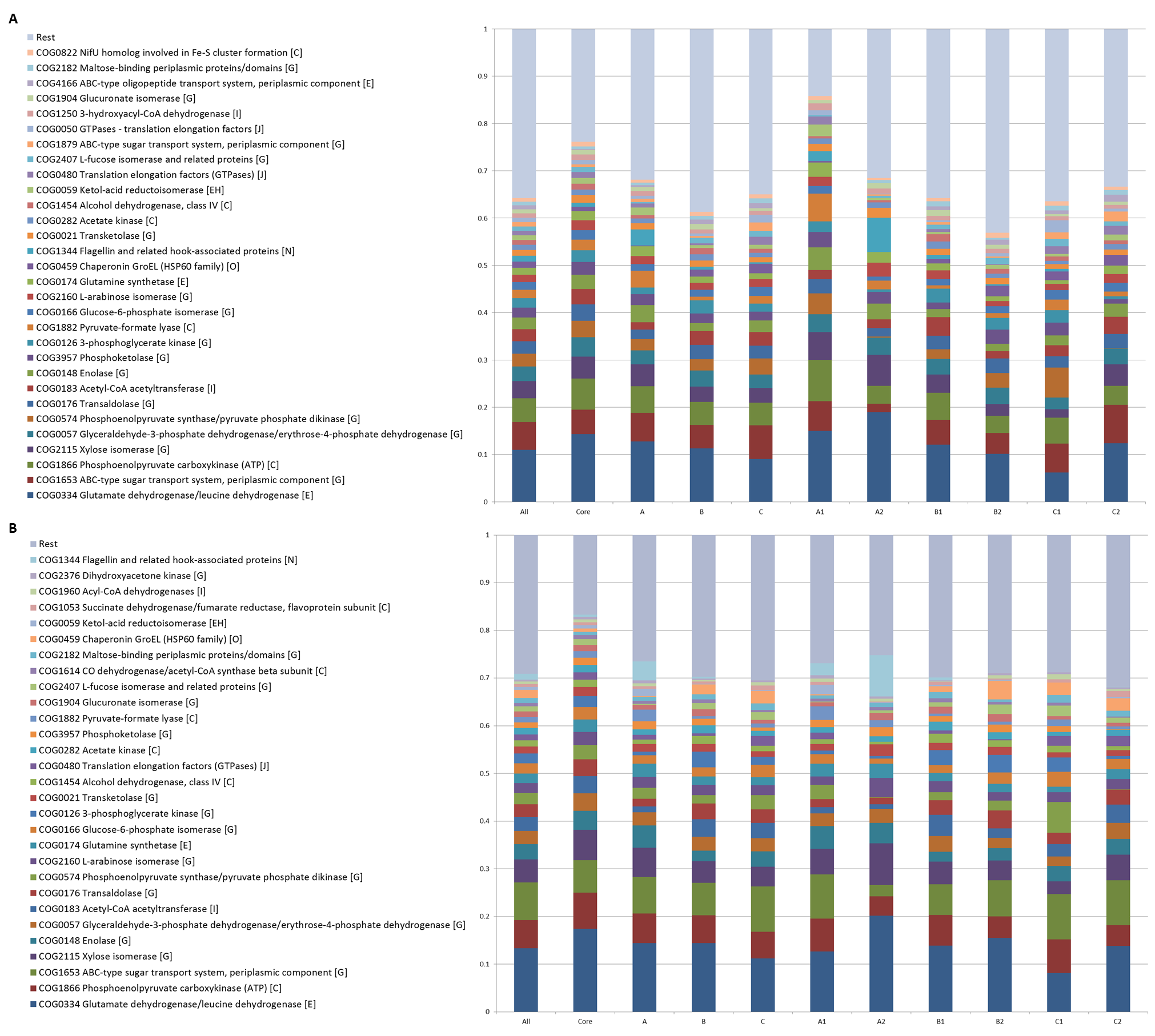

Supplement: Figure S4 — Functional comparison. A compositional view of the most abundant COGs (based on MS/MS identifications over all measurements, A; and over all AMP measurements, B) is shown. From left to right the bars visualize the COG distribution of all subjects and time points (all), the core, the subjects (A, B, C) and for each time point per subject (A1, A2, B1, B2, C1, C2). (TIF) [file pone.0029913.s004.tif]

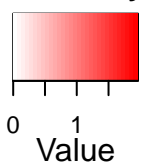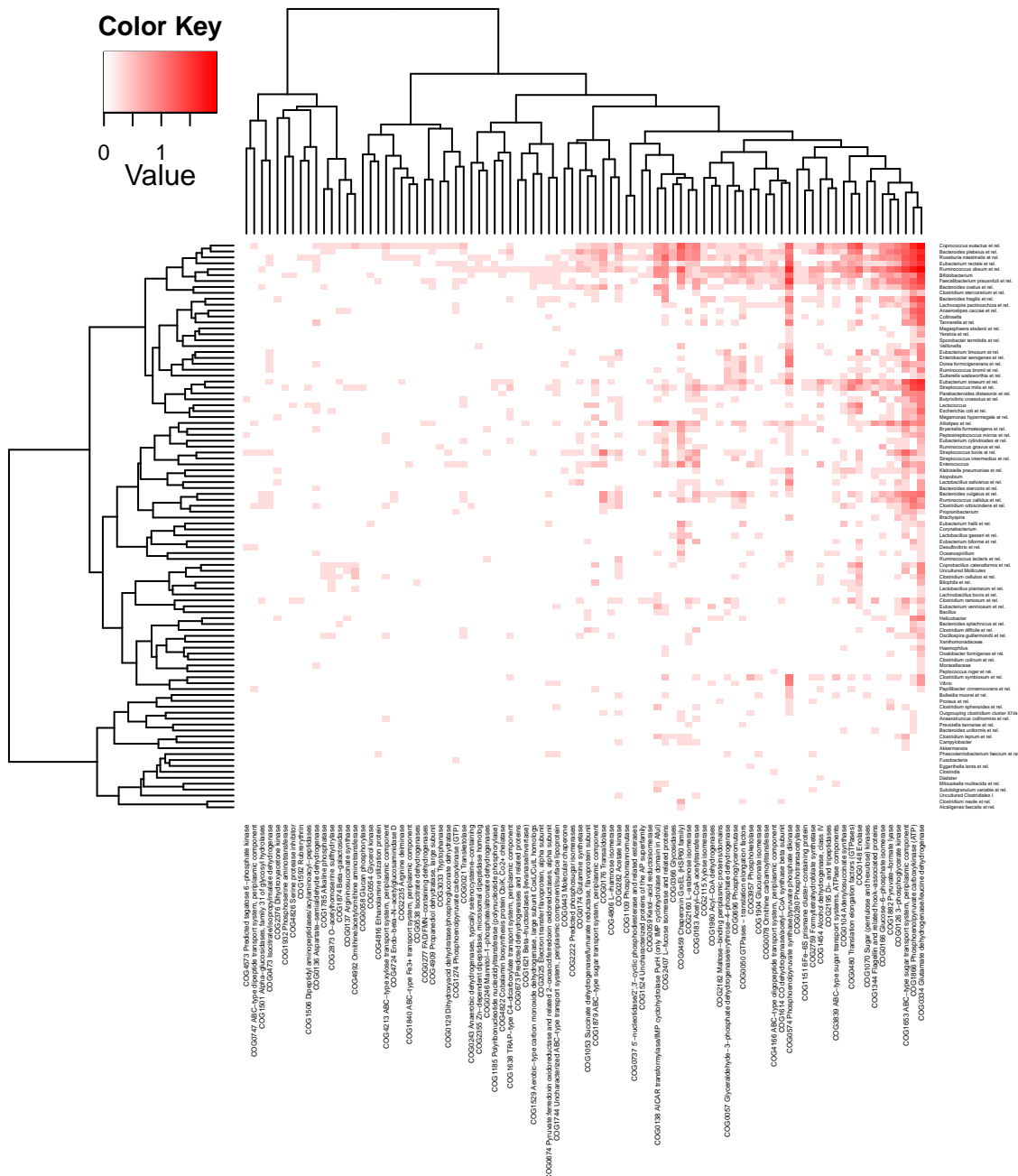

Supplement: Figure S5 — Heatmap of significant associations between metaproteome and HITChip data. Each row represents a genus-level taxon and each column represents a COG group. The colour key indicates the log10 of the number of significant associations between proteins and the enriched genus level groups. (PDF) [file pone.0029913.s005.pdf]
