# Supplementary material for: Comparative Metaproteomics and Diversity Analysis of Human Intestinal Microbiota Testifies for Its Temporal Stability and Expression of Core Functions
Source: PLoS One. 2012 Jan 18;7(1):e29913. doi: 10.1371/journal.pone.0029913 (PMC3261163; doi:10.1371/journal.pone.0029913)
Supplement: Figure S2 — Protein intensities per sample. Protein intensities in abundant metaproteome AMP over three subjects and two time points. Triangles indicate the region in the heatmap where the respective protein is found. Red scale: intensities ≥105; white: intensity ≤105. (PDF) [file pone.0029913.s002.pdf]

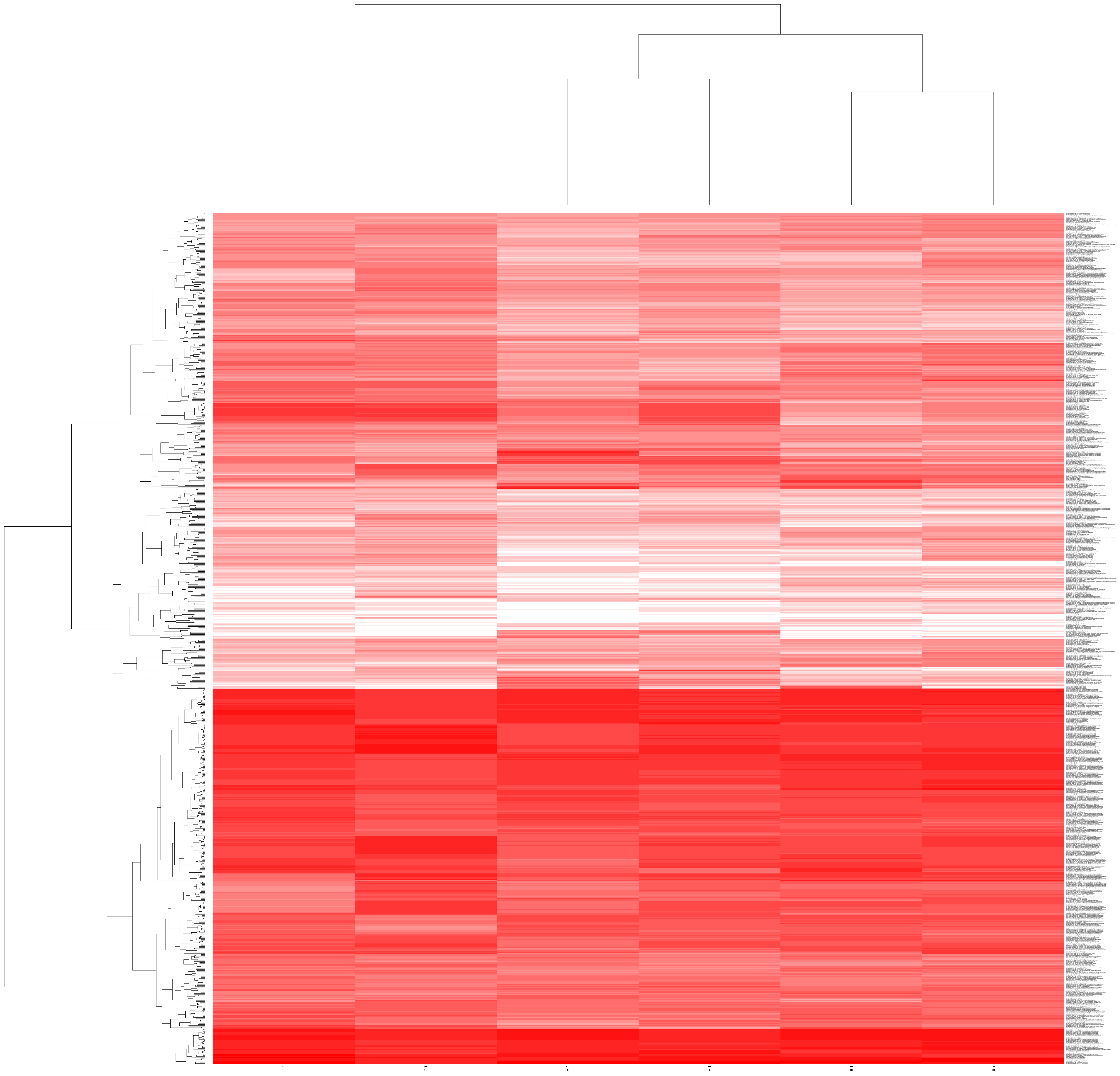
C.2
C.1
A.2
A.1
B.1
B.2
